# Aryl hydrocarbon receptor regulates histone deacetylase 8 expression to repress tumor suppressive activity in hepatocellular carcinoma

**DOI:** 10.18632/oncotarget.9841

**Published:** 2016-06-06

**Authors:** Li-Ting Wang, Shyh-Shin Chiou, Chee-Yin Chai, Edward Hsi, Shen-Nien Wang, Shau-Ku Huang, Shih-Hsien Hsu

**Affiliations:** ^1^ Graduate Institute of Medicine, College of Medicine, Kaohsiung Medical University, Kaohsiung 807, Taiwan; ^2^ Department of Pediatrics, Faculty of Medicine, College of Medicine, Kaohsiung Medical University, Kaohsiung 807, Taiwan; ^3^ Division of Hematology-Oncology, Department of Pediatrics, Kaohsiung Medical University Hospital, Kaohsiung 807, Taiwan; ^4^ Department of Pathology, Faculty of Medicine, College of Medicine, Kaohsiung Medical University, Kaohsiung 807, Taiwan; ^5^ Department of Genome Medicine, College of Medicine, Kaohsiung Medical University, Kaohsiung 807, Taiwan; ^6^ Division of Hepatobiliary Surgery, Department of Surgery, Kaohsiung Medical University, Kaohsiung 807, Taiwan; ^7^ Department of Surgery, faculty of Medicine, Kaohsiung Medical University Hospital, Kaohsiung 807, Taiwan; ^8^ Division of Environmental Health and Occupational Medicine, National Health Research Institutes, Zhunan 115, Taiwan; ^9^ Center for Environmental Medicine, Kaohsiung Medical University, Kaohsiung 807, Taiwan; ^10^ Center of Infectious Disease and Cancer Research (CICAR), Kaohsiung Medical University, Kaohsiung 807, Taiwan

**Keywords:** AHR, HDAC8, hepatocellular carcinoma (HCC)

## Abstract

Histone deacetylase 8 (HDAC8), a unique member of class I histone deacetylases, shows remarkable correlation with advanced disease stage and multiple malignant tumors However, little is known about the contribution of HDAC8 to the tumorigenesis of hepatocellular carcinoma (HCC). The present study investigated the expression of HDAC8 regulated by the aryl hydrocarbon receptor (AHR) in HCC cell lines and tissues, and the roles of HDAC8 overexpression in cell proliferation, including potentially underlying mechanisms. We assessed the correlation between the clinic-pathological parameters and the expression of AHR and HDAC8. Further, we analyzed the *AHR* siRNA transfection and HDAC8 inhibitors to explore the functions of HDAC8 in HCC progression *in vitro* and *in vivo*. In a panel of 289 HCC patients, HDAC8 was shown to be highly correlated with AHR expression at both mRNA and protein levels. HCC patients with high AHR expression showed a shorter survival time than that with low AHR expression. We then found that the expression of both *AHR* and *HDAC8* was significantly upregulated in both HCC cell lines and tumor tissues compared to human normal hepatocytes and matched non-tumor tissues. Furthermore, HDAC8 inhibition remarkably inhibited hepatoma cell proliferation and transformation activity via upregulation of RB1 *in vitro* and *in vivo*. Our data revealed an important role of the AHR-HDAC8 axis in promoting HCC tumorigenesis, thus identifying HDAC8 as a potential therapeutic target for HCC treatment.

## INTRODUCTION

The liver is the major organ involved in the metabolism of xenobiotics resulting from environmental pollution or dietary intake. A multitude of xenobiotics and their metabolites have detrimental and tumorigenic effects on hepatocytes and may cause liver tumor formation [1]. The aryl hydrocarbon receptor (AHR), a unique cellular chemical sensor in most organs, is a cytosolic basic helix–loop–helix/Per–Arnt–Sim transcriptional factor [2]. It can be activated by numerous environmental pollutants, including polycyclic aromatic hydrocarbons and a diverse set of endogenous metabolites [3, 4]. AHR resides in the cytoplasm in its resting state and upon encountering ligands, it is translocated to the nucleus where it heterodimerizes with the AHR nuclear translocator (ARNT) [5] and activates the expression of a battery of genes containing specific DNA-enhancer sequences, which are known as xenobiotic-responsive elements (XREs) [6]. These downstream genes include those encoding phase I and phase II enzymes [7] that are involved in the cellular detoxification process, where toxic and carcinogenic metabolites can be generated, which may play an important role in multiple stages of tumor progression in humans and experimental models [2, 7]. In addition to cellular detoxification, AHR is known to regulate signal transduction pathways involved in cellular metabolism, proliferation, differentiation, and apoptosis. Disruption of the AHR cellular functions is also associated with the expression of various diseases [7, 8]. Several studies have shown that dysregulation of AHR in hepatocytes leads to steatosis and aberrant cholesterol metabolism [9–11], and increased expression of AHR and its binding partner, ARNT, has been noted in hepatocellular carcinoma (HCC) [12]. However, the pathogenic mechanisms and the possible prognostic value of *AHR* expression in HCC are yet to be elucidated.

Histone deacetylases (HDACs), a family of enzymes with the ability to remove acetyl groups from lysine on histones and other proteins to repress downstream gene expression by wrapping the DNA more tightly, substantially contribute to the onset and progression of human diseases [13, 14]. In humans, HDACs are grouped in four classes of proteins: class I, IIa, IIb, III, and IV [15–17]. HDAC8, a class I zinc-dependent HDAC, typically induces histone deacetylation and represses gene transcription [18, 19]. HDAC8 is restricted to specific cell types exhibiting smooth muscle differentiation in normal human tissues. Loss of *HDAC8* activity has been shown to result in increased SMC3 acetylation and inefficient dissolution of cohesin complexes [20]. Aberrant upregulation of *HDAC8* was suggested to be correlated with NAFLD-associated HCC development [21]. Although HDAC8 has been shown to promote growth of numerous cancer types and contribute to poor prognosis in childhood neuroblastoma [22–24], the molecular actions of HDAC8 in cancer remained poorly defined.

In this study, we provide evidences that suggest a plausible mechanism linking AHR and HCC via targeting of *HDAC8*, which promotes tumor cell growth and may restrain the expression of RB1 tumor suppressors in HCCs.

## RESULTS

### HDAC8 showed tumor-specific expression pattern correlated with HCC clinical outcome

**Figure 1:**
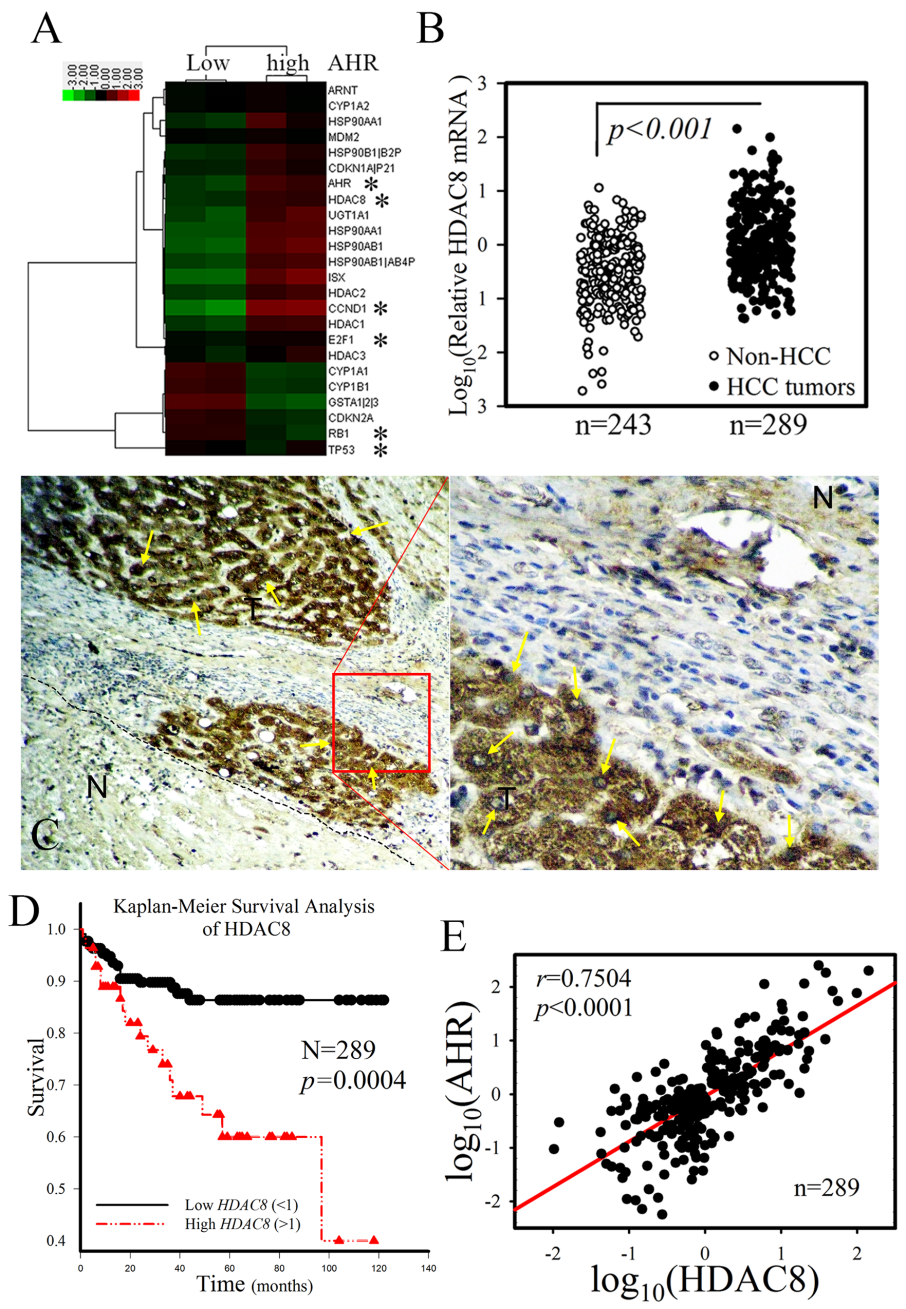
HDAC8 showed a tumor-specific expression pattern and strong correlation with the clinical outcome of HCC patients **A.** Heat map analysis of cDNA microarray data of “high“ AHR HCC tumors compared with data from adjacent samples of healthy liver tissue. High HDAC8 mRNA expression was detected in HCC tumors with high AHR expression. **B.** HDAC8 mRNA was overexpressed in HCC tumor samples compared with non-HCC liver samples. **C.** HDAC8 expression showed a tumor-specific pattern in HCC tumor samples. HDAC8, brown; HDAC8 (nuclei), yellow arrow; N, adjacent healthy liver tissue. **D.** High HDAC8 mRNA expression in HCC is associated with a shorter survival time in HCC patients than that associated with low HDAC8 mRNA expression (Kaplan–Meier survival analysis). **E.** HDAC8 expression showed a high correlation with AHR mRNA expression. The HDAC8 mRNA expression in HCC tumors with high AHR mRNA expression was significantly higher than that in tumors with low AHR mRNA expression.

AHR, a major chemical sensor receptor, is known to regulate signal transduction pathways involved in cellular metabolism, proliferation, differentiation, and apoptosis [3, 4]. Disruption of the cellular functions of AHR is also associated with the expression of various diseases, including HCC [25]. The underlying regulatory mechanisms of tumorigenesis remain unclear. To investigate the mechanism of the oncogenic action of AHR, we analyzed the gene expression patterns in paired samples of tumors from the high and low *AHR* group by microarray analysis. The expression of epigenetic genes, *HDAC8*, was elevated in high-*AHR* HCC tumors but decreased in the tumor suppressor genes *RB1* and *p53* (Figure 1A). To investigate the correlation and clinical outcome between the expression of *AHR* and *HDAC8*, the mRNAs for these genes were examined in a panel of 289 paired HCC tumor samples (tumor paired with neighbor “healthy” liver tissues). Analysis of the expression patterns of *HDAC8* in various tissue samples first showed that *HDAC8* mRNA expression in HCC liver samples was significantly upregulated as compared with that in non-HCC patients (Figure 1B; *p* = 0.0008, one-way ANOVA), while the expression pattern of *HDAC8* in HCC samples varied greatly. In fact, analysis of the dichotomized group with the *HDAC8* expression at the top 20 percentile (*n* = 56; “high *HDAC8*”) showed distinct clinical features compared with those of the remaining HCC patients (*n* = 230; “low *HDAC8*”). As shown in Table 1, when the two groups and the non-HCC subjects were compared, significant differences were observed in the levels of albumin (*p* = 0.0062) and lymph vascular invasion (*p* = 0.0163) as well as in tumor size (*p* = 0.002), tumor number (*p* = 0.0429), and tumor grade (*p* = 0.001), but not in age, sex, or the levels of GOT, alkaline phosphatase, triglyceride, g-GT, AC sugar, bilirubin, and cholesterol (Table 1). Further, HDAC8 expression in tumor samples showed a tumor-specific expression pattern in HCC tumor masses (indicated in brown; T) compared with the adjacent healthy liver tissues (N) and negative mouse IgG (Supplementary Figure S1), and HDAC8 expression was detected in both the cytoplasm and nucleus (yellow arrow) of tumor cells (Figure 1C). The analysis of the survival curves of HCC patients showed a significantly shorter survival time after surgical resection for patients in high *HDAC8* groups than in the low *HDAC8* expression groups (Figure 1D, p = 0.0004).

**Table 1:** Baseline characteristics of 289 hepatocellular carcinoma (HCC) patients and 243 non-hepatocellular carcinoma patients

Group	Non-HCC (n=243) (%)	*HDAC8* mRNA (Low, n=230)(%)	*HDAC8* mRNA (High; n=59)(%)	*p-value*
Age (mean(SD))	61.6±5.04	57.9±2.3	59.9±1.4	0.6851
Sex Male Female	154(63.37) 89(36.63)	164(71.30) 66(28.70)	48(81.36) 11(18.64)	0.6017 ^#^
GOT (U/L) <40 40≦ <100 100≦	214(88.06) 16(6.58) 13(5.36)	94(40.87) 102(44.35) 34(14.78)	19(32.20) 26(44.07) 14(23.73)	0.2670^#^
GPT (U/L) <40 40≦<100 100≦	216(88.89) 14(5.76) 13(5.35)	85(36.96) 106(46.09) 39(16.95)	21(35.59) 26(44.07) 12(20.34)	0.8844^#^
Albumin (mg/dL) <4.5 ≧4.5	219(90.12) 24(9.88)	195(84.78) 35(15.22)	57(96.61) 2(3.39)	0.0062*^#^
α-Fetoprotein (ng/mL) <20 ≧20	218(89.72) 25(10.28)	130(56.52) 100(43.48)	34(57.63) 25(42.37)	0.8909^#^
Bilit 1.5< ≧1.5	132(54.32) 111 (45.68)	189(82.17) 41(17.83)	50(84.75) 9(15.25)	0.8223^#^
Lymphovascular invasion No Yes	243(100)0	165(71.74) 65(28.26)	32(57.14) 27(45.76)	0.0163*^#^
Size(cm) <2.5 2.5≦		82(35.65) 148(64.35)	8(13.56) 51(86.44)	0.0020*
Number of tumors 1 1		181 (78.70) 49(21.30)	39(66.10) 20(33.90)	0.0429*
Modified TNM I II III(IIIA and IIIB)		153(66.52) 61(26.52) 16(6.96)	23(38.98) 20(33.90) 16(27.12)	<0.001*

#p values were calculated by Fisher exact test

*High HDAC8 mRNA expression patients: HDAC8 mRNA expression in tumor part were higher three point six folds (>3.6 folds) than adjacent normal part liver tissue*.

Low HDAC8 mRNA expression patients: HDAC8 mRNA expression in tumor part were lesser three point six folds (<3.6 folds) than adjacent normal part liver tissue. (cut point by survival ROC curve)

*p<0.05.

*Patients:289HCC patients from two medical centers [Chung Ho Memorial Hospital (255HCC) and Changhua Christian Hospital (34 HCC)] were enrolled into the HDAC8 cohort study from July 2007 to July 2015*.

### HDAC8 mRNA expression correlated with AHR expression

**Figure 2:**
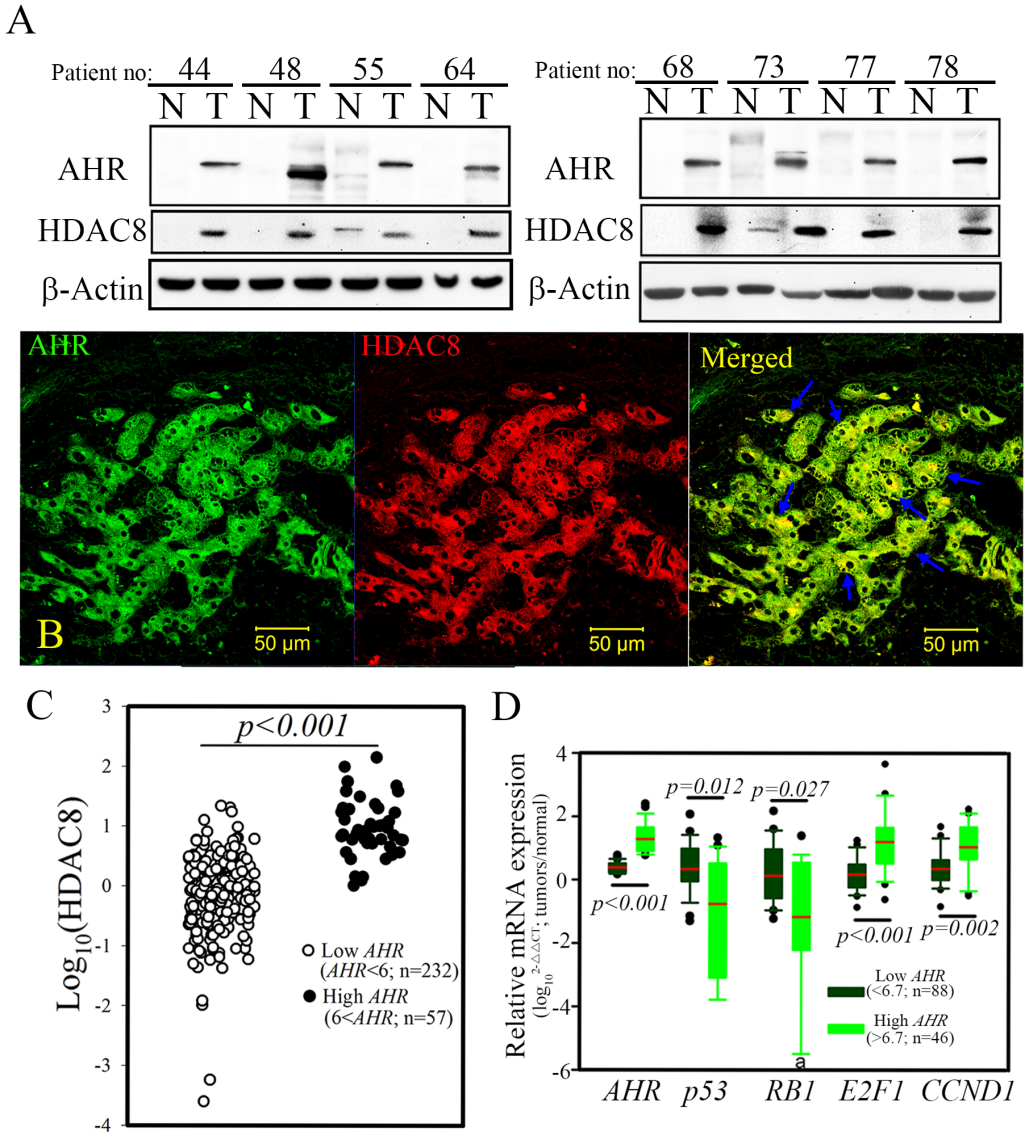
Ectopic HDAC8 expression showed a high correlation with AHR in hepatocellular carcinoma (HCC) **A.** Overexpression of HDAC8 protein was detected in HCC patients with high AHR mRNA expression. **B.** The expression pattern of HDAC8 showed significant co-localization with AHR in tumor cells of HCC patients. HDAC8, red; AHR, green. **C.** High levels of HDAC8 mRNA were detected in HCC patients exhibiting high AHR mRNA expression. **D.** High AHR expression coupled with high proto-oncogenes (CCND1 and E2F1) expression but with lower tumor suppressor gene (p53 and RB1) expression in HCC patients. a, six samples from the high AHR group could not be detected.

The expression of *HDAC8* mRNA strongly correlated with the expression of *AHR* in HCC patients (Person's correlation coefficient, *r* = 0.7504, *p* < 0.001; Figure 1E). In 8 randomly selected HCC samples obtained from patients in the high *AHR* group, the increased *HDAC8* expression correlated with the expression pattern of *AHR* when compared with those of paired tumor-adjacent, normal tissues (Figure 2A). The expression correlation between AHR and HDAC8 was further verified by immunofluorescence staining of HCC patient tumor tissue. High level of AHR expression was noted in tumor cells co-expressing high level of HDAC8 (Figure 2B). *HDAC8* mRNA expression in tumors of the “high *AHR*” group was significantly higher than that in the “low *AH*R” group (Figure 2C). Analysis of the dichotomized group (“high *AHR*” *vs*. “low *AHR*” expression) placed the expression of *HDAC8*, *p53*, *RB1*, *E2F1*, and *CCND1* in the top 25 percentile (*n* = 57; “high *AHR*”). *HDAC8* expression and expression of *CCND1* and *E2F1* in tumors of the “high *AHR*” group were significantly higher than in the “low *AHR*” group (*n* = 232; “low *AHR*;” Figure 2D). *RB1* and *p53* expression showed opposite correlation in HCC tumors (Figure 2D).

### AHR-ARNT1 complex genetically activated HDAC8 expression

**Figure 3:**
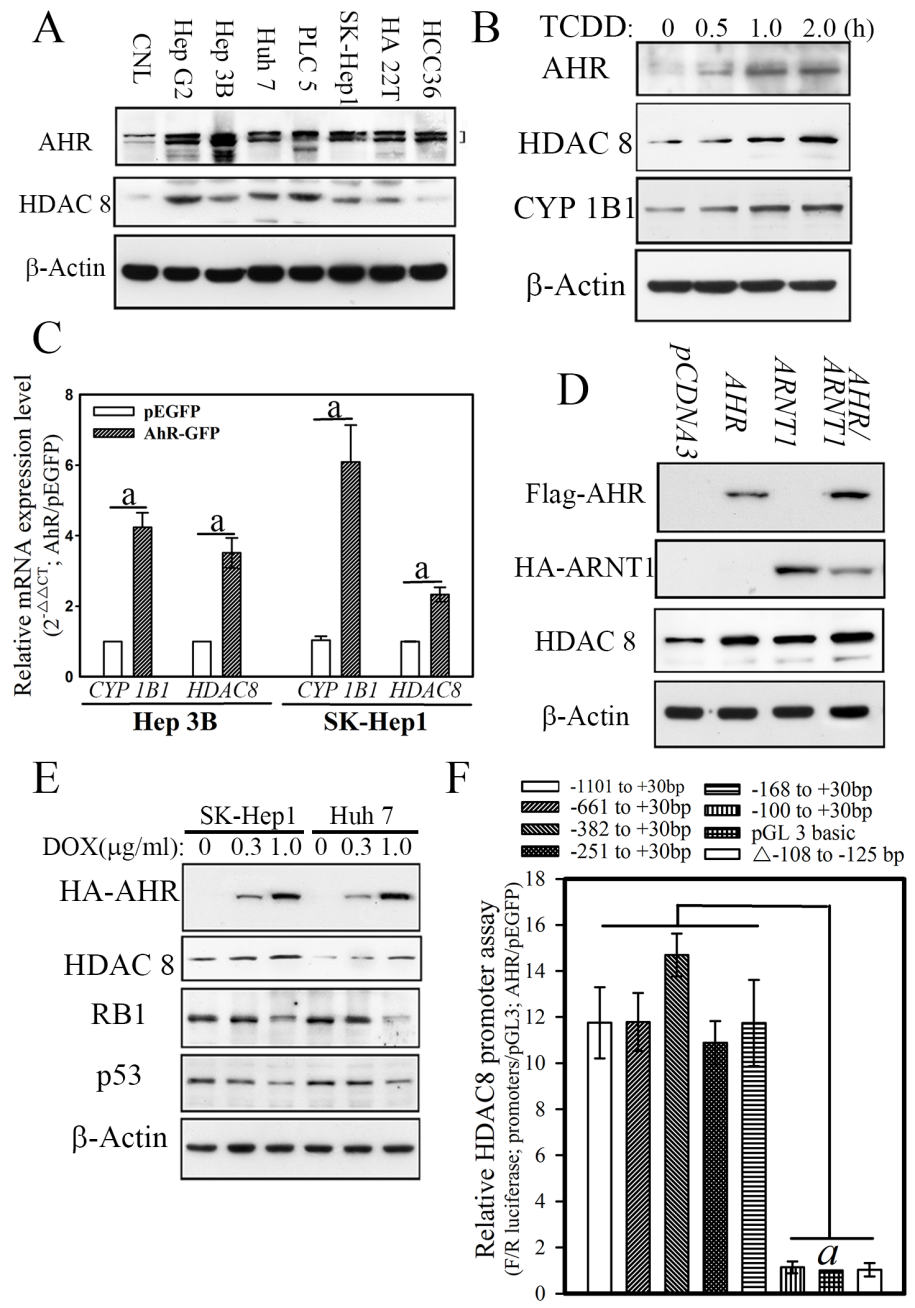
AHR directly activated cellular HDAC8 expression via the AHR–ARNT complex in hepatoma cells a, p < 0.001. **A.** AHR expression in hepatoma cell lines showed strong correlation with the expression of HDAC8 in Hep G2, Hep 3B, SK-Hep1, Huh 7, PLC/PRF/5, and HA22T are HCC cell lines. **B.** AHR activation induced by TCDD increased cellular CYP1B1 and HDAC8 protein levels in hepatoma cells. **C.** Forced AHR expression increased the mRNA expression of the downstream genes CYP1B1 and HDAC8. **D.** Forced AHR expression increased the protein expression of the downstream genes CYP1B1 and HDAC8. Forced AHR and ARNT1 showed an additive effect on HDAC8 expression. **E.** AHR expression induced by doxycycline increased HDAC8 expression but decreased p53 and RB1 expression in hepatoma cells. **F.** AHR activated luciferase activities driven by the HDAC8 promoter until promoter regions less than −168 bp.

To explore the potential functional relationship between AHR and HDAC8, analyses of the expression patterns of AHR and HDAC8 were first examined in hepatoma cell lines. Analyses of seven hepatoma cell lines (Hep G2, Hep 3B, SK-Hep1, Huh 7, PLC/PRF/5, HA22T, and HCC36) revealed that AHR and HDAC8 were highly expressed in all of the hepatoma cell lines as compared with expression levels in normal hepatocytes (Chang normal liver cells, CNL; Figure 3A). Moreover, when hepatoma cells (SK-Hep1) were treated with TCDD, a prototypical AHR ligand, expressions of HDAC8 and the classical AHR target gene *CYP1B1* were increased in hepatoma cells in a time-dependent manner, both at protein level (Figure 3B). To determine whether AHR activated *HDAC8* via a genetic mechanism, we transfected an *AHR* expression construct into hepatoma cells, and the mRNA and protein levels of *HDAC8* were measured by real-time PCR and western blotting, respectively, in *AHR*-overexpressing cells. As shown in Figure 3C and 3D, forced *AHR* overexpression enhanced mRNA and protein expression of *CYP1B1* and *HDAC8* in both hepatoma cell lines. The protein level of *HDAC8* was also increased when *AHR-GFP* overexpression (induced by a Tet-on system) was induced by different concentrations of doxycycline (0.3 and 1 μg/mL) for 8 h (Figure 3E). Interestingly, reduction in the expression levels of two tumor suppressors, RB1 and p53, was also noted in *AHR*-overexpressing hepatoma cells. This was consistent with the finding that in HCC clinical samples, the expression levels of *RB1* and *p53* were significantly lower in the “high *AHR* group” in comparison to those in the “low *AHR* group,” suggesting the existence of an inverse relationship between high and low *AHR* expression of these two tumor suppressors.

**Figure 4:**
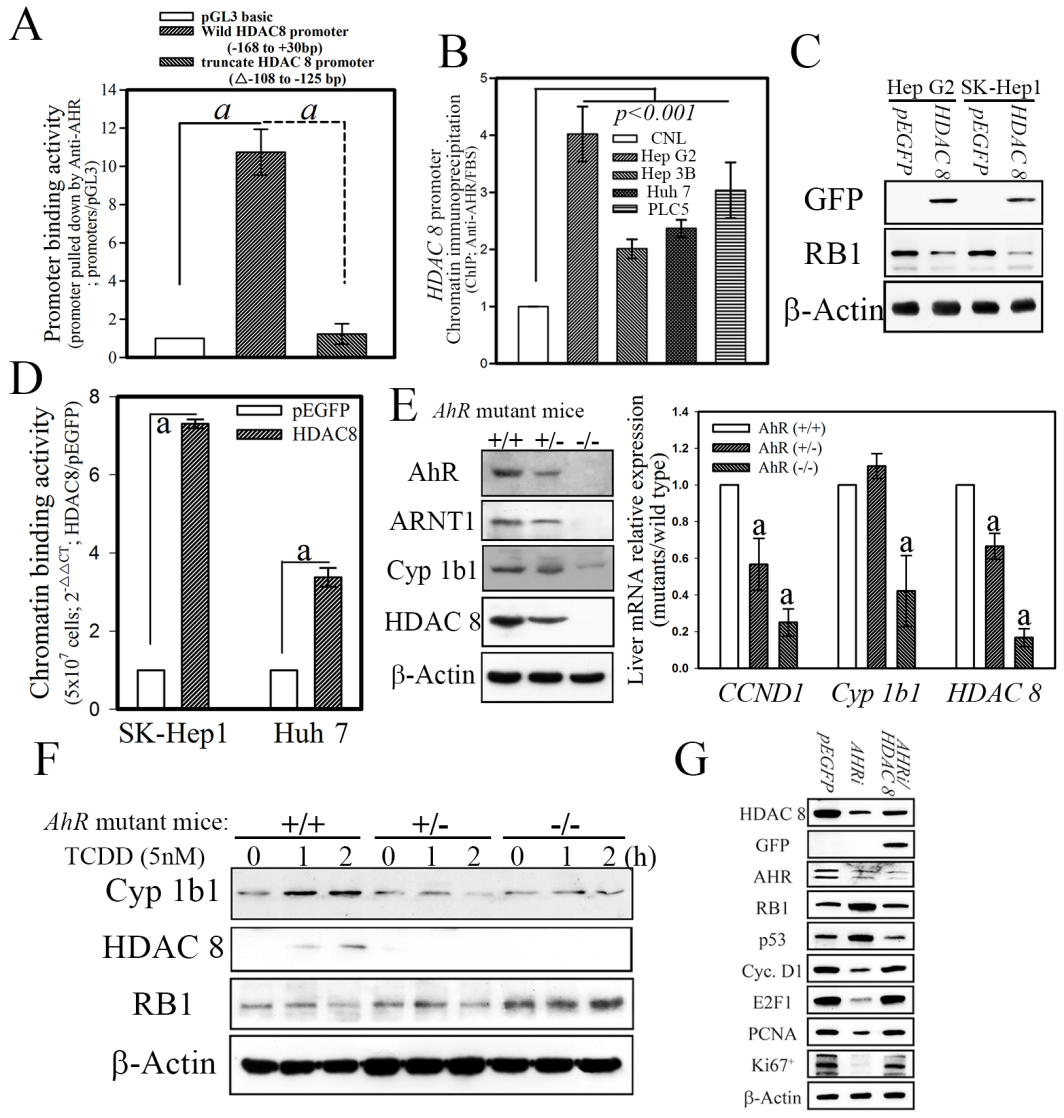
AHR physiologically regulates HDAC8 expression in normal mouse hepatocytes a, p < 0.001. **A.** The HDAC8 promoter with the −108 to −125 bp deletion abolished the promoter binding activity of AHR. **B.** Chromatin HDAC8 promoter binding activity showed an AHR-dependent increase in hepatoma cells. The HDAC8 promoter binding activity of the AHR complex in hepatoma cells determined with ChIP *in vivo*. **C.** Forced HDAC8 downregulated RB1 expression. **D.** Forced HDAC8 expression in hepatoma cells enhanced the chromatin binding activity of HDAC8 on the RB1 promoter. **E.** HDAC8 expression in hepatocytes was abrogated in AhR-null mice. **F.** The ablation of HDAC8 expression in hepatocytes showed involvement of AHR-dependent regulation. **G.** The hepatoma cells (SK-Hep1) transfected with HDAC8 showed to downregulate the enhancement on tumor suppressors, p53 and RB1, expression induced by cells transfected with AHR shRNAi.

To further explore and verify the regulatory effects of AHR on *HDAC8* expression, we used a promoter assay and chromatin immunoprecipitation (ChIP) to analyze the potential transcriptional regulatory elements to which AHR binds in the *HDAC8* promoter. A *HDAC8* promoter with serial deletions was inserted into a luciferase expression construct to identify the regulatory promoter region that is controlled by AHR (Figure 3F). AHR significantly increased *HDAC8* promoter–driven luciferase activity (11.89 ± 1.74) of the expression constructs with a promoter sequence longer than 168 bp (Figure 3F). Analysis of the *HDAC8* promoter region between positions −168 and +30 identified one classical XRE (T/GNGCGTGA/CG/CA) in the locus comprising −108 to −125. The transactivation effect of AHR on the XRE element of the *HDAC8* promoter was further confirmed by a promoter binding assay. As shown in Figure 4A, the *HDAC8* promoter regions (positions −168 to +30) were pulled down by an anti-AHR polyclonal antibody in SK-Hep1 hepatoma cell lines transfected with *AHR*. In contrast, the *HDAC8* promoter region was not pulled down if XRE was deleted (Figure 4A). The AHR binding to *HDAC8* promoter regions *in vivo* were analyzed using ChIP analysis. The *HDAC8* promoter region between −168 and +30 bp was pulled down by the anti-AHR antibody in hepatoma cells with high HDAC8 expression (Figure 4B).

### HDAC8 downregulated RB1 expression via direct promoter binding

To explore the potential functional relationship between HDAC8 and RB1, analyses of the expression patterns of HDAC8 and RB1 were first examined in hepatoma cell lines. An *HDAC 8* expression construct was transfected into hepatoma cells, and the levels of RB1 were measured by Western blotting in *HDAC 8*-overexpressing cells. As shown in Figure 4C, forced *HDAC 8* overexpression suppressed protein expression of RB1 in hepatoma cell lines. Also, the *RB1* promoter regions (positions −250 to +30) were pulled downed by the anti-HDAC8 polyclonal antibody in SK-Hep1 hepatoma cell lines transfected with *GFP-tagged HDAC8* (Figure 4D). Our data showed that the induction of AHR activated cellular expression of HDAC8 and consequently suppressed RB1 expression in hepatoma cells. However, it remained unclear whether the expression of HDAC8 occurs in an AHR-dependent manner under physiological conditions. To address this question, the expression of Arnt1, Cyp1b1, and Hdac8 was analyzed in hepatocytes from *AhR*-wild type (*AhR* ^+/+^) or–null (*AhR*^−/−^) mice. Results revealed dramatically reduced protein and mRNA levels for all parameters in *AhR*-null hepatocytes compared with those of *AhR*^+/+^ or *AhR*^+/−^ cells (Figure 4E). In fact, hepatocytes from *AhR*^+/−^ heterozygotes also showed lower levels of gene expression when compared with those of *AhR*-wild type cells (Figure 4E). To test if the inhibition of RB1 and HDAC8 expression was directly affected by *AhR*, hepatocytes isolated from *AhR*-wild type and *AhR*-null mice were treated with dioxin. As shown in Figure 4F, increased HDAC8, but decreased Rb1, expression was found in dioxin-treated *AhR*-wild type primary hepatocytes but not in those from *AhR*-null mice. The modulation regulatory effect of RB1 by HDAC 8 was finally determined in hepatoma cells with ectopic *HDAC 8* expression but transfected with *AHR* shRNAi. The hepatoma cells (SK-Hep1) with ectopic HDAC 8 expression reversed the enhancing effects of AHR knockdown on the expression of RB1 and p53 (Figure 4G). These results indicated that *HDAC8* are direct physiological targets of *AhR* and that Rb1 in turn regulates cellular proliferation as a consequence of AhR activation.

### The AHR-HDAC8 axis regulated tumor proliferation and transformation in vitro and in vivo

**Figure 5:**
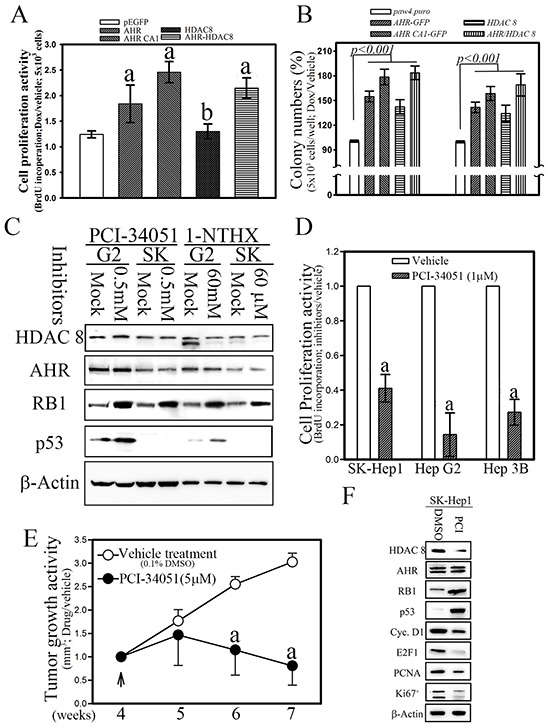
Ectopic expression of AHR and HDAC8 is essential for cell proliferation and transformation in hepatoma cells b, p < 0.05 and a, p < 0.001. **A.** Ectopic expression of AHR and HDAC8 increased cell proliferation. CA1, constitutively activated AHR mutant. **B.** Ectopic expression of AHR and HDAC8 significantly enhanced cellular transformation activities. **C.** The HDAC8-specific inhibitors PCI-34051 (1 μM) and 1-NTHX (50 μM) increased the expression of the tumor suppressors RB1 and p53. **D.** The HDAC8-specific inhibitor PCI-34051 repressed the cell proliferation activity of hepatoma cells *in vitro*. **E.** and **F.** The HDAC8-specific inhibitor PCI-34051 repressed cell proliferation and tumor growth activity of hepatoma cells with HDAC8 over-expression in nude mice (7.4 μg/kg body weight; twice per day).

To explore the potential tumorigenic activity of the AHR-HDAC8 axis, an inducible expression system overexpressing *AHR*, a constitutively activated AHR mutant (*CA1*) [10], and *HDAC8* was tested in two HCC cell lines (Hep 3B and SK Hep1). After treatment of the cells with doxycycline (1 µg/mL) for 8 h, overexpression of AHR or the CA1 mutant significantly enhanced BrdU incorporation by 47.9% and 97.8%, respectively, as compared with those transfected with the empty-vector control (Figure 5A). The hepatoma cells with HDAC8 expression showed a lesser degree of enhancement (30%) of BrdU incorporation than those overexpressing AHR. Hepatoma cells with both AHR and HDAC8 expression showed an additive effect on cell proliferation activity. Enhancement of cellular transformation activity by *AHR*, *CA1*, and *HDAC8* in HCC cells was observed in an independent soft agar assay (AIG), wherein overexpressed *AHR*, *CA1* mutant, and *HDAC8* significantly increased colony formation by 54%, 78%, and 42%, respectively, after induction by doxycycline (Figure 5B). The hepatoma cells exhibiting both *AHR* and *HDAC8* expression showed a more pronounced additive effect on transformation activity than that of *AHR* or *HDAC8* alone.

To further evaluate the role of AHR-HDAC8 signaling in the proliferation and tumorigenesis of HCC, hepatoma cells treated with HDAC8-specific inhibitors, PCI-34051, and 1-Naphthohydroxamic acid (1-NTHX) [26, 27], showed enhanced expression of RB1 and p53, as well as a lower level of proliferative activity (Figure 5C and 5D). The tumor growth *in vivo* of hepatoma cells was also reduced in nude mice treated with the HDAC8-specific inhibitor PCI-34051(5 μM) for four weeks (Figure 5E), and the tumor mass in mice treated with PCI-34051 showed lower levels of proliferation marker (Cyclin D1, E2F1, PCNA and Ki 67^+^) expression than those treated with DMSO (0.1%) (Figure 5F) [28]. These results indicated that AHR and its target, HDAC8, play an important role in terms of promoting tumorigenesis of HCC.

## DISCUSSION

AHR, a unique chemical sensor activated by a large number of xenobiotics, has been considered a major regulator of xenobiotic-induced carcinogenesis, but its role in the processes of tumor initiation and development remains to be elucidated. In this study, we showed that the inactivation of tumor suppressor *RB1* occurred by activation of AHR-HDAC8-axis signaling. In a panel of 289 HCC patients, *HDAC8* was shown to be highly correlated with *AHR* expression at both mRNA and protein levels. Genetically, AHR activated *HDAC8* expression via direct binding to the *HDAC8* promoter region and consequently downregulated tumor suppressor *RB1* expression. These results clearly show why chronic exposure of toxic environmental molecules may result in the development of hepatic neoplasia or malignant tumors.

Cancer is primarily an environmental disease with 90%–95% and 5%–10% of the cases being attributed to environmental and genetic factors, respectively [29]. Despite extensive research, environmental risk factors of cancer remain largely unknown. Many studies have shown that dysregulation of AHR in hepatocytes leads to steatosis and aberrant cholesterol metabolism [10], and increased expression of *AHR* has been noted in HCC [12], suggesting its potential pro-tumorigenic activity; however, the tumorigenic activity of AHR is still unclear. In this study, we provided further evidence that *AHR* expression influences HCC tumorigenicity by suppressing the expression of tumor suppressor *RB1*. This difference in genetic outcomes between the “low” and “high” *AHR* groups appeared to be determined by the change in expression levels of tumor the suppressor genes *p53* and *RB1* and of those of the proto-oncogenes *HDAC8*, *CCND1*, and *E2F1*. Evidence from our clinical data and analysis of tumor cells showed that high *AHR* expression directly enhanced its downstream gene-*HDAC8* expression, which led to low expression of tumor suppressors (*p53* and *RB1*); however, it was associated with higher levels of expression for proto-oncogenes (*CCND1* and *E2F1*) *in vitro* and *in vivo*. The results provided mechanistic evidence that the tumorigenic activation induced by high *AHR* expression is involved in HCC formation, suggesting a potential target for HCC therapy.

Inactivation of tumor suppressor activity of e.g., *RB1* and *p53* is the most frequent hallmark of cancer, including HCC [28, 30, 31]; however, the detailed regulatory mechanisms are still largely unknown. *RB1*, a classic tumor suppressor, is frequently inactivated in many malignances. In general, according to cancer genome studies, *RB1* was genetically inactivated in about 22% of patients with prostate cancer [32]. Evidence also indicates that the RB pathway is functionally inactivated in most cases of HCC by genetic, epigenetic, and/or viral mechanisms [33]; the activation of the RB pathway in a mouse model may prevent HCC development by maintaining the quiescence of adult liver progenitor cells [28, 34]. However, as evidenced by SNP analysis, genetic mutations of the *RB1* gene are relatively rare and few studies were focused on the inactivation of RB signaling in HCC. Recently, a systematic screen for the identification of imprinted genes deregulated in human HCC revealed that *RB1* shows imprint abnormalities in a high proportion of primary patient samples [35, 36]. Forty percent of the HCC specimens (16/40) showed hyper- or hypo-methylation at the CpG island in intron 2 of the *RB1* gene [35]. In this study, we first showed that, beside methylation, promoter deacetylation of the *RB1* gene induced by HDAC8 represents another potential important mechanism for *RB1* inactivation in human HCC, complementing already well-described molecular defects. These results provide advanced epigenetic evidence of acetylation in *RB1* expression, a potential therapeutic target in HCC.

In summary, we demonstrated that AHR regulated cell proliferation and tumorigenesis by directly targeting and activating *HDAC8* expression in hepatoma cells. The strong correlation of mRNA and protein levels between AHR and HDAC8 in patients with HCC underscores the important regulatory role of the AHR signaling axis in HCC progression, suggesting its potential utility as a new therapeutic target and for developing preventive measures in HCC.

## MATERIALS AND METHODS

### Patients

This study enrolled 289 patients with hepatocellular carcinoma (HCC) from July 2007 to July 2015 from two medical centers (Chung Ho Memorial Hospital [255 patients] and Changhua Christian Hospital [34 patients]) with hepatitis B virus (HBV) and/or hepatitis C virus (HCV) infection and 243 non-HCC patients, of whom 85 were infected with HBV or HCV and 68 were not infected with either virus. Of these patients, 289 had adequate follow-up data for detailed analysis. The study of human subjects was approved by the Institutional Review Board of Kaohsiung Medical University (KMUHIRB-20130052; Kaohsiung, Taiwan).

### Animals and cell culture

Male BALB/c nu/nu mice were obtained from the National Laboratory of Animal Breeding and Research Center (Taipei, Taiwan) and housed according to the protocols of the Animal Center, Kaohsiung Medical University, Kaohsiung, Taiwan [37]. Normal hepatocyte cells (Chang normal liver cells; CNL) and human hepatoma cell lines (HepG2, Hep3B, SK-Hep1, Huh7, PLC/PRF/5, HCC 36 and HA22T) were obtained from American Type Culture Collection (Manassas, VA, USA) and maintained according to the manufacturer's protocol [38].

### Plasmids, cell lines, and other materials

Full-length *AHR* cDNA was amplified using PCR from a human testis cDNA library (GIBCO/BRL). The *AHR* cDNA and a mutant *CA1* were each subcloned into the *pEGFP/C1* (pCDNA3/HA) vector (Clontech) or a Tet-on system (*pAW4.puro*, NCFPB, Taiwan) to express a GFP (HA)-tagged AHR. The *PLKO.1.puro* or *.neo* vector was used as a backbone for shRNAi constructs targeting *AHR* and *HDAC8* (NCFPB, Taiwan). SK-Hep1, Hep G2, Hep 3B, HA 22T, PLC cell lines were sub cultured and maintained according to American Type Culture Collection (ATCC) protocols. Transfection was performed using the Lipofectamine transfection kit (GIBCO/BRL).

### Western blotting and immunohistochemical analysis

Western blotting and immunohistochemical (fluorescence) staining were performed as previously described [37, 38]. The primary antibodies used in this study were AHR (RPT9), HDAC8 (GeneTex), RB1, p53, Cyclin D1, PCNA, ARNT1, CYP1B1 and E2F1 (1:200), actin polyclonal antibodies (1:5000 dilution; Sigma–Aldrich), HA and GFP monoclonal antibodies (1:200 dilution; Upstate, NY, USA), FITC-conjugated anti-rabbit IgG, rhodamine-conjugated anti-mouse IgG, alkaline phosphatase-conjugated anti-rabbit IgG antibody (1:500 dilution; Jackson ImmunoResearch Laboratories), and Ki67 goat polyclonal antibody (1:200 dilution; Santa Cruz Biotechnology) [28]. All of the experiments were repeated at least three times, and staining with relevant control antibodies was performed in parallel and in all cases no signals were seen (for example, Supplementary Figure S1).

### Luciferase reporter assays

The *HDAC8* promoter (between positions −1101 and +30 bp) was cloned from human placenta genomic DNA and was used to construct a pGL3 luciferase reporter plasmid [28, 38]. The expression constructs and two reporter constructs, pSV40-Rluc and pGL3-*HDAC8*/Fire luciferase (Promega), were co-transfected with *pEGFP-AHR* into 2 × 10^5^ Hep 3B cells. The cells were harvested 16 h after the transfection, and the relative luciferase activity was measured according to the manufacturer's instructions. All data are expressed as the mean ± SD of at least three experiments.

### Chromatin immunoprecipitation (ChIP) assays

Chromatin immunoprecipitation (ChIP) assays were performed as described previously [38]. All data are expressed as the mean ± SD of at least three experiments. An *HDAC8* promoter fragment was amplified with the following primers: Primer 1, 5′-TTC ACT GGG CGT CCG CAG AG-3′ and Primer 2, 5′-TGC CCC AAG CCT CCG CGA TC-3′. An *RB1* promoter fragment was amplified with the following primers: Primer 1, 5′-TTG CCA TTG CTG CTG TCA C-3′ and Primer 2, 5′-CGT CCT CCC AAA CCA TTC A-3′.

### Real-time PCR

The expression of *AHR* and *HDAC8* mRNA in hepatoma cells and cells from cancer patients were quantified using the SYBR Green Quantitative RT-PCR kit (Invitrogen) as described previously. Total RNA was extracted from the tumor mass using TRIzol reagent (Invitrogen) and transcribed into cDNA (Invitrogen) for PCR amplification using a 7900HT Thermocycler (Applied Biosystems Inc.). All procedures and data analyses were performed according to the manufacturer's instructions. Data on cells transfected with an empty *pEGFP* vector and samples from healthy subjects and drug-treated patients were analyzed and compared. All data are expressed as the mean ± SD of at least three experiments.

### Anchorage-independent growth assays

Cells (10^4^ or 5 × 10^3^) in 1 mL of culture medium were mixed with an equal volume of 0.6% top agar and plated onto 60-mm culture dishes with 0.5% bottom agar [37, 38]. The plates were incubated at 37 °C for 2 weeks. Colonies were visualized by staining with 0.05% crystal violet acetate, and only those larger than 0.5 mm were counted. The culture medium was replaced every 3 days. All data are expressed as the mean ± SD of at least three experiments.

### Assessment of the tumorigenic properties of *HDAC8* in nude mice

Male BALB/c nu/nu mice were inoculated (s.c. injection) with 10^6^ of either scrambled vector or *HDAC8-GFP*-transfected overexpression cells on both sides of the back (10 mice per group) [37, 38]. Tumor size was measured using a caliper once or twice a week. Tumor volume was estimated according to the formula: volume (cm^3^) = (L × W^2^)/2, where L and W represent the length and width of the tumor, respectively. Tumor sizes were presented as means ± SD.

### Statistical analysis

The quantitative variables are presented as means ± SD. The statistical significance of differences was determined using a two-sample *t*-test. Pearson's correlation analysis was used to examine the relationship between expression levels of *AHR* and *HDAC8*. Statistical analysis of categorical variables was performed using chi-squared analysis, one-way analysis of variance (ANOVA), and Fisher's exact test. Differences with a *p* value < 0.05 were considered statistically significant.

## SUPPLEMENTARY MATERIALS FIGURE


