# Correction: Aryl hydrocarbon receptor regulates histone deacetylase 8 expression to repress tumor suppressive activity in hepatocellular carcinoma

**DOI:** 10.18632/oncotarget.26547

**Published:** 2018-12-28

**Authors:** Li-Ting Wang, Shyh-Shin Chiou, Chee-Yin Chai, Edward Hsi, Shen-Nien Wang, Shau-Ku Huang, Shih-Hsien Hsu

**Affiliations:** ^1^ Graduate Institute of Medicine, College of Medicine, Kaohsiung Medical University, Kaohsiung 807, Taiwan; ^2^ Department of Pediatrics, Faculty of Medicine, College of Medicine, Kaohsiung Medical University, Kaohsiung 807, Taiwan; ^3^ Division of Hematology-Oncology, Department of Pediatrics, Kaohsiung Medical University Hospital, Kaohsiung 807, Taiwan; ^4^ Department of Pathology, Faculty of Medicine, College of Medicine, Kaohsiung Medical University, Kaohsiung 807, Taiwan; ^5^ Department of Genome Medicine, College of Medicine, Kaohsiung Medical University, Kaohsiung 807, Taiwan; ^6^ Division of Hepatobiliary Surgery, Department of Surgery, Kaohsiung Medical University, Kaohsiung 807, Taiwan; ^7^ Department of Surgery, faculty of Medicine, Kaohsiung Medical University Hospital, Kaohsiung 807, Taiwan; ^8^ Division of Environmental Health and Occupational Medicine, National Health Research Institutes, Zhunan 115, Taiwan; ^9^ Center for Environmental Medicine, Kaohsiung Medical University, Kaohsiung 807, Taiwan; ^10^ Center of Infectious Disease and Cancer Research (CICAR), Kaohsiung Medical University, Kaohsiung 807, Taiwan

**This article has been corrected:** Figures 2A and 3B were displayed incorrectly. The proper figure images are given below. The authors declare that these corrections do not change the results or conclusions of this paper.

**Figure 2:**
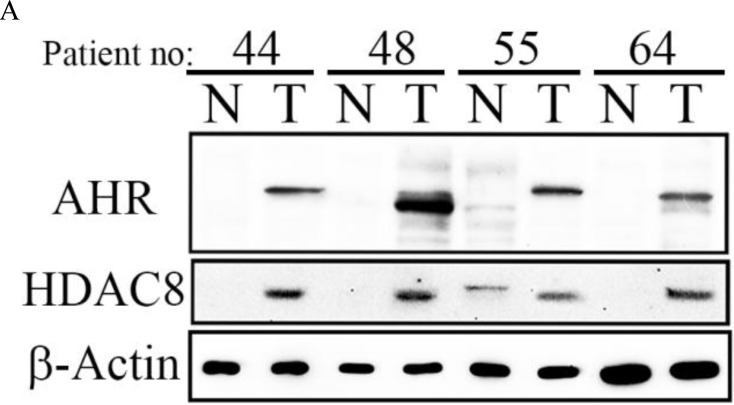
Ectopic HDAC8 expression showed a high correlation with AHR in hepatocellular carcinoma (HCC) **A.** Overexpression of HDAC8 protein was detected in HCC patients with high AHR mRNA expression.

**Figure 3:**
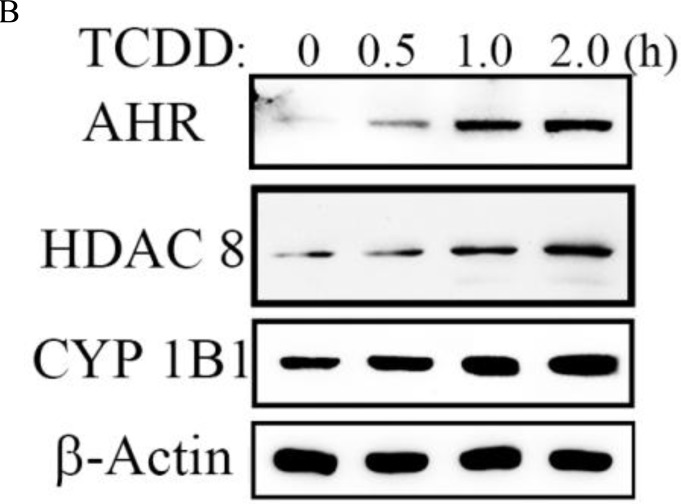
AHR directly activated cellular HDAC8 expression via the AHR–ARNT complex in hepatoma cells **B.** AHR activation induced by TCDD increased cellular CYP1B1 and HDAC8 protein levels in hepatoma cells.

Original article: Oncotarget. 2017; 8:7489-7501. https://doi.org/10.18632/oncotarget.9841

