# Supplementary material for: Aryl hydrocarbon receptor regulates histone deacetylase 8 expression to repress tumor suppressive activity in hepatocellular carcinoma
Source: Oncotarget. 2016 Jun 6;8(5):7489–501. doi: 10.18632/oncotarget.9841 (PMC5352337; doi:10.18632/oncotarget.9841)
Supplement: Supplementary file 1 [file oncotarget-08-7489-s001.pdf]

Aryl hydrocarbon receptor regulates histone deacetylase 8 expression to repress tumor suppressive activity in hepatocellular carcinoma

SUPPLEMENTARY FIGURE

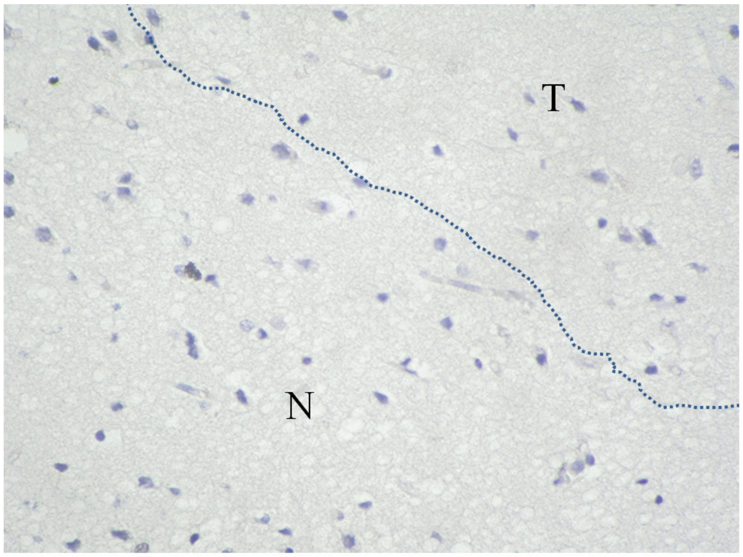

Supplementary Figure S1: The immunohistochemistry staining of HCC tumors by mouse IgG. N, normal liver cell; T, tumors.
